# Prostate cancer with neuroendocrine differentiation – case report

**Published:** 2012-03-05

**Authors:** G Glück, M Mihai, R Stoica, R Andrei, I Sinescu

**Affiliations:** Center of Urology and Renal Transplantation, Fundeni Clinical Institute, Bucharest, Romania; Departament of Pathology, Fundeni Clinical Institute, Bucharest, Romania

**Keywords:** synaptophysin, Cromogranin A, immunohistochemistry

## Abstract

**Rationale: **About 95% of prostate cancers are adenocarcinoamas. Depending on the detection method used, neuroendocrine cells are found in 10% to 100% of prostate cancer specimens.

**Objective: **A 64-year-old patient was diagnosed in 2006 with adenocarcinoma of the prostate, PSA 4.1 ng/ml, Gleason 6, T3b, positive PSA immunohistochemistry.

**Methods and results: **The patient was started on hormone therapy: orchidectomy followed by flutamide 750 mg/day for three years, and underwent radiotherapy 6400 R. The patient was asymptomatic for three years. In 2009, the patient complained of perineal and rectal pain, but the PSA remained normal. In 2010, the patient underwent TUR of the prostate for acute urinary retention. Pathological exam revealed Gleason 8 adenocarcinoma of the prostate (different pathologist suggested Gleason 9) and foci of neuroendocrine cells. Immunohistochemistry detected 15-20% positivity for Cromogranin A and 10% for synaptophysin. The patient developed multiple liver metastases in October 2010 and underwent five cycles of etoposide, carboplatin. The patient died of liver failure in March 2011.

**Discussion: **Regarding prevalence, neuroendocrine differentiation is the second phenotype after prostate adenocarcinoma, but still remains undiagnosed. It is resistant to radiation therapy and chemotherapy. Detection of the neuroendocrine differentiation is recommended during the clinical, biochemical, histopathological and immunohistochemical follow up of prostate cancer patients treated by EBRT and / or androgen deprivation.

**Abbreviations** CT computerized tomography; MRI magnetic rezonance imaging; NE neuroendocrine; NSAID nonsteroidal anti-inflamatory drug; PSA prostatic specific antigen; EAU European Association of Urology; PET Positron Emission Tomography; EBRT external beam radioyherapy

## Introduction

Prostate cancer is one of the most common neoplasic diseases in male population. According to the National Cancer Institute, regarding prostate cancer, 217,730 new cases were diagnosed and 32,050 deaths were registered due to prostate cancer in 2010, in the United States. In 2008, there were approximately 338,000 cases in the European Union. In Romania, the incidence was of 32.2 (cases per 100,000 inhabitants) in the year 2006, compared to the United States, where the incidence was of 95.9 for Caucasian and 127.6 for Afro-American population (mortality was of 3.14/100.000).

## Case presentation

**Fig. 1:**
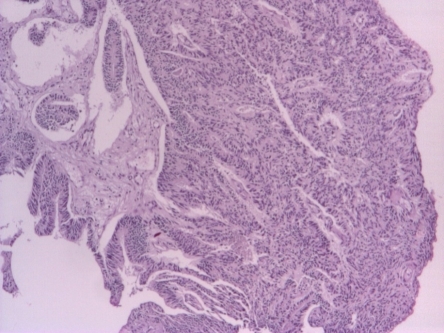
Prostate adenocarcinoma, Gleason score 6 (3+3), August 2006

 A 64-year-old male patient, with a single left kidney (history of right nephrectomy for benign condition), admitted in our Department, for episodes of hematuria, in August 2006, when, based on the clinical examination (rectal examination outlining a prostate of 4/4 cm with an endured left lobe), PSA of 4.1 ng/ml and transurethral prostate biopsy was diagnosed with prostate cancer - well-differentiated papillary tubulointerstitial adenocarcinoma, Gleason score was of 6 (3 +3). **Fig. 1**

MRI evaluation of the prostate revealed a prostate tumor with left seminal vesicle invasion (T3b) and small pelvic lymph-nodes. Patient’s choice was surgical orchidectomy and antiandrogen hormonal therapy (flutamide 3x250 mg / day). It was locally irradiated with 6400 Rad.

Immunohistochemical examination from 16.10.2006 revealed an intensely positive PSA, tubulo-papillary and cribriform adenocarcinoma of the prostate (Gleason 6). The patient underwent the follow-up protocol according to the EAU guidelines until 2009, being asymptomatic, with a PSA level of 0,003 ng/ml, and with no postvoid residual.

From February 2009 the patient accused extremely intense pain in the perineum and rectum. PSA was of 0.003 ng/ml. Abdominal and pelvic CT and bone scan were within normal limits. He completely emptied the bladder. Under the usual treatment with analgesics and NSAIDs, the pain was controlled, until February 2010. Digital rectal examination revealed local minimal changes of the prostate. Pelvic MRI showed a 4,5 cm tumor prostate with left extracapsular invasion, the infiltration of the mezorectal fascia, of the left levator ani muscle and a 26 mm lesion in the left femoral head. 

**Fig. 2:**
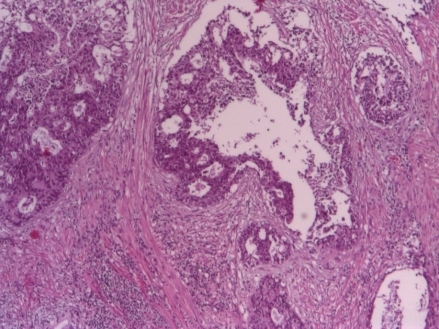
Adenocarcinoma of the prostate Gleason 9 (4+5) 2010

The patient underwent a PET scan, which showed a metabolically active prostate tumor and the left femoral lesion. In May 2010, the patient developed urinary retention managed by TURP: pathological exam revealed adenocarcinoma of the prostate Gleason score was 8 (3 +5), **Fig. 2**

**Fig. 3:**
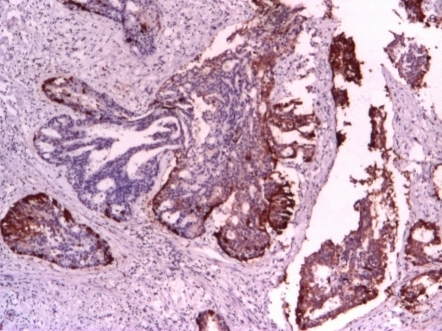
CROMOGRANIN 4x(2010)

**Fig. 4:**
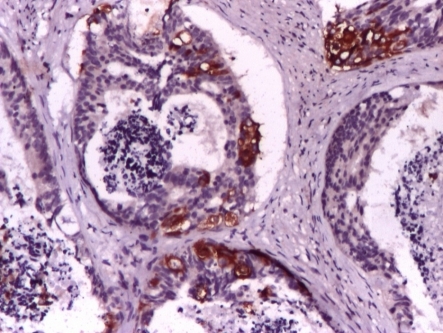
SYNAPTOPHYSIN 10x(2010)

The patient asked for a second opinion in an urology clinic in Turkey. Repeated MRI confirmed the extracapsular invasion. Ultrasound: bladder capacity of 175 ml, prostate 55cc, postvoid residual 45 ml. Paraffin blocks sent for a second opinion: prostate adenocarcinoma, Gleason score 3 +5 (**Fig. 2**), neuroendocrine differentiation, with positivity for Cromogranin (**Fig. 3**) and synaptophysin (**Fig. 4**). 

**Fig. 5:**
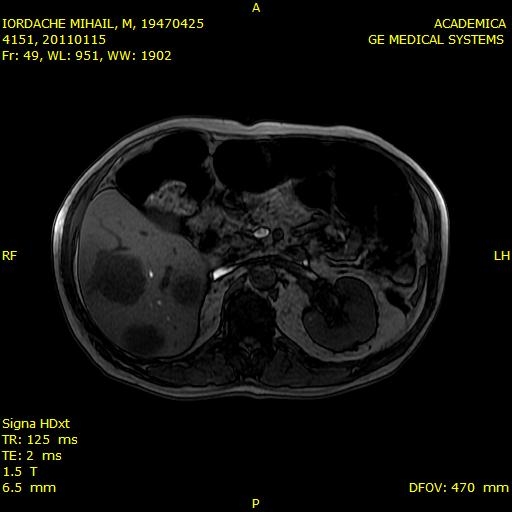
MRI revealed multiple formations in the liver parenchyma (October 2010)

The serum Cromogranin was of 199 micrograms / ml (normal up to 98) and serotonin 5-hydroxy-indolacetic acid was normal. The treatment with Carboplatin and Etoposide was initiated. Painful symptoms were remitted. The patient underwent five cycles of chemotherapy, but developed severe neutropenia. In October 2010, the MRI revealed multiple masses in the liver parenchyma, varying between 10 and 20 mm. (**Fig. 5**). 

Since November 2010 he was treated with 20 mg of Sandostatin LAR per month, then with Farmarubicine.

In February 2011, he developed an upper urinary tract dilatation with acute renal failure (serum creatinine reached 6 mg/dL and oliguria). Percutaneous left nephrostomy was inserted. The diuresis resumed and the values of creatinine reached normal levels. In March 2011, he died from liver failure.

## Discussion

Cellular elements of the prostate, are found on a base membrane of ductal and acinar secreting cells, basal cells, among which are the stem cells and the neuroendocrine cells belonging to the APUD system [**1**] (amino precursor uptake decarboxylation), which can be opened to the gland lumen and in contact with neural dendrites, or it may be closed (no communication with the lumen). [**2-4**]

Prostate cancers are 95% adenocarcinomas. Depending on the technique used to detect neuroendocrine cells (NE) they are from 10% to 100% present in prostate cancers. [**3,5**] Histopathological forms are: 

- Small cell carcinomas, [**6,7**] - Carcinoids [**8**] - Foci of neuroendocrine neoplasic cells in prostatic adenocarcinoma.[**4,6**] This is of major importance because prostate cancers with neuroendocrine differentiation have a poor prognosis (35% survival at 2 years) compared with cases where there are no NE cells (97% survival at 2 years). [**9,10**]

One of the most important things to note, is that the differentiation process of these neuroendocrine cells can appear over time. [**11-13**]

The hormone dependency of the prostate adenocarcinoma is known since 1941. With androgen deprivation therapy, a regression for a period of time is achieved, after that the phenomenon of hormone-resistance or hormone independence appears. [**14,15**]

It seems that the NE cells are the most in this period, these cells displaying apoptotic activity of only 0.16%, in other words they are an immortal cell population in patients with prostate cancer. [**3,16,17**] They produce substances such as somatostatin, bombesin and serotonin, involved in cell growth and metastasis. Among the characteristic tissue and serum markers, are the cromogranin A, [**10,18**] serotonin and the synaptophysin. [**19,20**]

**Fig. 6:**
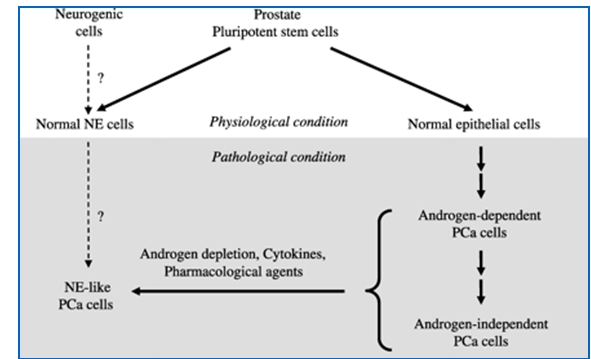
Neuroendocrine-like cells in prostate cancer: neuroendocrine transdifferentiation of prostate adenocarcinoma cells. [**16**]

This patient was diagnosed with adenocarcinoma of the prostate in 2006, PSA 4.1 ng / ml, Gleason 6, T3b and positive PSA immunohistochemistry. The patient was irradiated up to 6400 Rad! Ablative hormone therapy (orchidectomy) and antiandrogen (flutamide 750 mg / day) was established. For 3 years he was asymptomatic. In 2009, he accused perineal and rectal pain but without PSA rise. In 2010, the prostate was re-resected and prostate adenocarcinoma Gleason score 8 and foci of neuroendocrine cells were diagnosed. Serum Cromogranin A was of 199 micrograms/L (compared with 98- maximum admitted value), serotonin was 85.9 micrograms/L (compared to 117-190 mg/L), 5-hydroxy-indolacetic acid 5.6 mg/24 hours (N=2-9). Immunohistochemistry showed 15-20% positivity for Cromogranin A and 10% for synaptophysin. In October 2010, the patient developed multiple liver metastases. He underwent five courses of Carboplatin with etoposide, but, because of the severe adverse events (febrile neutropenia) the chemotherapy was suspended and monthly treatment with 20 mg of Sandostatin was instituted. He died due to liver failure in March 2011.

## Conclusions

Neuroendocrine differentiation characterizes the second phenotype by prevalence in comparison with the prostate adenocarcinoma, but remains under diagnosed. NE tumor cells are androgen-insensitive and have a mitogenic effect on adjacent tumor cells (exocrine).

They are resistant to irradiation and chemotherapy. The monitoring and diagnosis of NE differentiation is recommended in clinical, laboratory, histopathological and immunohistochemical monitoring of the patients with prostate cancer treated by irradiation and/or androgen deprivation therapy. 
